# Mediating role of attenuated physiological arousal on the association between psychopathic traits and fairness norm violation

**DOI:** 10.1038/s41598-019-54676-z

**Published:** 2019-12-02

**Authors:** Takahiro Osumi

**Affiliations:** Department of Psychology, Hiroshima Shudo University, Hiroshima, Japan

**Keywords:** Decision, Personality, Social behaviour, Morality, Human behaviour

## Abstract

The low-fear model of primary psychopathy has been supported by empirical findings such as attenuated physiological arousal in anticipation of threatening stimuli. The somatic marker hypothesis proposes that salient changes in the bodily state are processed as signals of whether a situation is good or bad and guide an individual to avoid potential adverse consequences. The present study aimed to elucidate the role that attenuated physiological arousal plays in the relationship between primary psychopathy and fairness norm violations both under the threat of punishment and under no potential for punishment. Primary psychopathy was associated with an attenuated skin conductance response prior to the choice of unfair monetary offers to another person, regardless of the potential for punishment. Attenuated skin conductance mediated the association between primary psychopathy and the choice of an unfair offer, especially in the no-punishment condition. However, in the punishment condition, primary psychopathy significantly predicted the choice of unfair offers even after controlling for the magnitude of skin conductance. The bodily response may have only a marginal effect on interpersonal decision-making under a threat of punishment. The present results suggest that the low-fear account of social norm violations as a function of primary psychopathy should be re-discussed.

## Introduction

In society, in general terms, if people exhibit behaviors that deviate from legal, moral, or conventional norms, they are supposed to be punished in the form of a criminal penalty or social sanction, and, at worst, lose things that are important for leading a social life. Nevertheless, psychopathic individuals frequently engage in violations of social norms^1^. Psychopathy is a group of personality traits including a lack of remorse and guilt, shallow affect, manipulativeness, egocentricity, stimulation seeking, impulsivity, and a lack of long-term goals^1,2^. A classical conceptualization distinguishes primary psychopathy from secondary psychopathy because they are associated with antisocial behaviors due to different etiological roots^3,4^. Whereas the behavioral problems of secondary psychopaths derive from neurotic conflicts, negative affect, and/or impulsivity, primary psychopaths exhibit antisocial acts in an unemotional manner. Lykken^5,6^ proposed that the core feature of primary psychopathy is a deficit in fear and/or anxiety. In this theory, it is assumed that a reduced aversive response to punishment makes psychopathic individuals more likely to engage in behavior that is linked to punishment. In support of Lykken’s low-fear model, individuals who score high in primary psychopathy in both incarcerated and community populations exhibit a reduced skin conductance response (SCR) selectively to aversive events, such as viewing unpleasant pictures^7^ and listening to unpleasant sounds^8,9^. Furthermore, they are less physiologically aroused by conditioned stimuli during fear conditioning tasks^5,10,11^ and in anticipation of aversive stimuli^8^. These findings suggest that deficient functioning of the biological defensive (fear) system, which is activated by threatening cues and promotes physiological states to avoid threats, underlies primary psychopathy^12^. However, it is still unclear whether a deficit in fear accounts for the association between primary psychopathy and social norm violations that are linked to punishment stimuli.

According to Damasio^13^, salient changes in somatic and autonomic activities spontaneously guide an individual to avoid threatening or risky environmental stimuli since bodily responses are processed as a signal of whether a situation is good or bad. This somatic marker hypothesis (SMH) is based on findings from laboratory studies using the Iowa gambling task (IGT), in which participants choose a card from four decks that are divided into two disadvantageous decks and two advantageous decks based on different amounts of monetary gain/loss and probability^14^. While participants who show an increased SCR prior to selection of a disadvantageous card avoid the disadvantageous risk, those who fail to generate an anticipatory SCR before choosing disadvantageous cards are likely to choose disadvantageous cards^14,15^. Such SCR results in the IGT have been generally replicated, but it remains unclear exactly what these SCRs represent^16^. Especially, it has been claimed that anticipatory SCRs in the IGT reflect, not the anticipation of long-term disadvantageous outcomes, but rather the increased variance in the immediate rewards and punishments offered by a deck^17^. Furthermore, there is a debate regarding whether the IGT is a valid test for somatic marker functioning^18^, since IGT performance is associated with awareness of the reward/punishment schedule^19^ and executive functions^20^. However, despite the difficulty of interpreting the behavioral and physiological results in the IGT, there is increasing evidence that physiological responses and the afferent detection of bodily states are related to intuitive decision-making^21,22^.

Attenuated physiological arousal in anticipation of punishment stimuli seems likely to cause a deficiency in putative somatic marker processing in individuals with primary psychopathy. According to the fact that anticipatory SCRs are increased with greater variance in immediate reward and punishment^17^, attenuated physiological arousal may serve as a bridge between primary psychopathy and increased decisions that carry a risk for receiving a high degree of punishment and a chance for gaining a high reward. To support this prediction, it would be helpful to investigate the relationship among psychopathic traits, physiological arousal, and risky decision-making. Nevertheless, the relationship between psychopathic traits and reduced physiological reactivity in anticipation of aversive events has been demonstrated independently of the decision-making process. Another potential issue is the ecological validity of the results from non-social experimental settings. In fact, the low-fear model of primary psychopathy was grounded on evidence from studies that used simple and basic aversive stimuli (electric shock, loud or noisy sounds, and pictures of threatening scenes). However, a meta-analysis revealed that psychopathy is less likely to be associated with deficits in the recognition of facial and vocal expressions of anger, which are considered to be social signals that individuals can use to predict the possibility of punishment^23^. Therefore, studies are needed to examine whether primary psychopathy attenuates responsivity in anticipation of punishment during social interactions.

The ultimatum game (UG)^24^ and the dictator game (DG)^25^ are widely used to study decision-making regarding whether or not to violate fairness norms in interpersonal interactions. In these tasks, a sum of money is divided between two players, and one player unilaterally decides how the money is to be distributed to him/herself and the second player. In the DG, both players are then assigned money based solely on the first player’s decision. According to these rules, if the first player is rational, they will offer no money to the second player. Nevertheless, participants who act as the first players are not completely rational, and instead voluntarily and altruistically distribute some amount of the stake to their partner (on average, about 30%)^26^. On the other hand, in the UG, the second player can decide whether to accept or reject the offer made by the first player. If the second player accepts the offer, then the deal goes forward. However, if the second player rejects the offer, then neither player receives any money. Rejections of unfair UG offers are generally considered to be punishment or revenge toward the first player who made the offer^27^, and are associated with negative emotions, especially anger^28,29^. Since offers in the UG are more likely to be fair than offers in the DG^30^, the performance of the first player in the UG may be affected by psychological factors, including emotions in response to the possibility of having low offers rejected. Thus, the UG may be useful for investigating the sensitivity to anticipated punishment during social interactions.

To date, most studies have indicated that primary psychopathy is associated with increased unfairness of offers in the DG in both forensic and community populations^31–34^. On the other hand, a few studies have examined the relationships between psychopathic traits and decision-making by the first player in the UG, with mixed results. One study did not find a difference in the unfairness of offers in the UG between offenders with primary psychopathy and non-psychopathic offenders, though the sample size was small^32^. In contrast, while limited to data collected using hypothetical scenarios, another study reported that the tendency for primary psychopathy in the general population predicted an increased likelihood to make unfair offers in the UG^34^. However, the proportion of unfairness in the UG was less than that in the DG regardless of the tendency in primary psychopathy. Thus, there is no clear-cut evidence that primary psychopathy attenuates the responsivity to anticipated punishments in social situations, at least on a behavioral level. These UG data raise a question about compatibility with a deficit in physiological responses that support the low-fear model of primary psychopathy.

The present study aimed to examine whether primary psychopathy moderates the responsivity to anticipated punishments in social situations, both behaviorally and physiologically. This study also aimed to statistically test the mediating role of attenuated physiological arousal on the association between psychopathic traits and fairness norm violations in both the punishment and no-punishment conditions. To this end, this study measured SCR before participants decided upon monetary amounts to be distributed to themselves and an anonymous partner in both the UG, where there is a potential for punishment (punishment condition), and the DG, where there is no potential for punishment (no-punishment condition). Since UG offers are more fair than DG offers^30^, it is presumed that an aversive response to a potential for punishment is an essential factor in suppressing unfair offers. If such an emotional response is represented as physiological arousal, participants might exhibit larger SCRs in the UG relative to the DG, especially prior to making unfair offers. However, based on the low-fear model^5,6^ and evidence for deficient functioning of the biological defensive system^12^, it is hypothesized that higher levels of primary psychopathy would be associated with increased fairness violations and reduced SCR prior to such decisions despite the potential for punishment. Moreover, if physiological arousal has the effect of avoiding risky options, a reduced SCR would mediate the association between primary psychopathy and fairness norm violations in the punishment condition.

In the DG, despite the lack of risk for receiving punishment from the partner, offers can be modulated for emotional reasons. In particular, the amounts of DG offers have been shown to be increased in relation to the feeling of guilt and the induction of empathic emotions for the predicted distress of others^35,36^. Following the SMH, bodily responses may be associated with such feelings that can promote the hesitation of unfair and self-interest behaviors. If this is the case, the association between psychopathic traits and increased unfair DG offers will be accounted for by a deficit in somatic marker functioning. Therefore, in the no-punishment condition, it is hypothesized that an attenuated SCR prior to the choice of unfair offers would play a mediating role on the association between psychopathic traits and unfair offers.

## Results

### Effects of punishment and psychopathic traits on the choice of unfair offers

Table 1 shows means and standard deviations for the scores of primary and secondary psychopathy on the Levenson self-report psychopathy scale (LSRP)^37^, which is a self-report questionnaire for assessing psychopathic traits in non-institutionalized populations. Table 1 also shows means and standard deviations for the choice ratios under each unfairness level (low: 50% and 40%; medium: 30% and 20%; high unfair offers: 10% and 0% of the stake for the partner), and the SCR magnitudes before participants chose such offers in the punishment and no-punishment conditions. Pearson’s correlation coefficients between these variables are presented in the Supplementary Table S1.

**Table 1:** Means and standard deviations for main variables (*N* = 35).

			M	SD
LSRP	Primary psychopathy		35.800	7.094
	Secondary psychopathy		21.971	3.960
Choice ratio	Punishment	Low unfair offers	0.695	0.277
		Medium unfair offers	0.603	0.130
		High unfair offers	0.368	0.253
	No-punishment	Low unfair offers	0.500	0.363
		Medium unfair offers	0.568	0.088
		High unfair offers	0.598	0.362
SCR (log[1 + μS)	Punishment	Low unfair offers	0.097	0.074
		Medium unfair offers	0.098	0.076
		High unfair offers	0.105	0.093
	No-punishment	Low unfair offers	0.096	0.084
		Medium unfair offers	0.088	0.074
		High unfair offers	0.090	0.074

LSRP: Levenson self-report psychopathy scale; SCR: skin conductance response.

Whether psychopathic traits modulate the effects of punishment on the behavioral choice of offers was tested. The frequencies of the choice of low, medium, and high unfair offers were not fully independent in the current experimental design. Because high unfair offers are more likely to be associated to higher potentials for punishment than low and medium unfair offers, only the frequency of high unfair offers was used as a dependent variable in a hierarchical linear model (HLM)^38^. Initially, the intraclass correlation (ICC) coefficient was calculated. When the ICC is more than trivial (i.e., greater than 10% of the total variance in the outcome), a hierarchical structure can be considered^39,40^. For the current data on the frequency of high unfair offers, the ICC coefficient was 0.35. Thus, 35% of the variance of the frequency of high unfair offers was between-individual, which provided evidence for a hierarchical structure. The results of HLM are shown in Table 2. A main effect of punishment was significant (*B* = −0.230, *p* < 0.001). In addition, there was a significant main effect of primary psychopathy (*B* = 0.019, *p* < 0.001). In contrast, the main effect of secondary psychopathy was not significant (*B* = −0.008, *p* = 0.35). A cross-level interaction between punishment and primary psychopathy was not found (*B* = −0.001, *p* = 0.89). Also, the interaction between punishment and secondary psychopathy was not statistically significant (*B* = −0.020, *p* = 0.07).

**Table 2:** Fixed effects for the HLM for predicting the choice of very unfair offers.

Level 1	Level 2	Unstandardized coefficient	*SE*	95% CI	*t*-value	*p*-value
Intercept	Intercept	0.483	0.040	0.401–0.565	12.010^***^	<0.001
	PP	0.019	0.004	0.011–0.027	4.648^***^	<0.001
	SP	−0.008	0.008	−0.025–0.009	−0.955	0.347
Punishment	Intercept	−0.230	0.049	−0.331–−0.130	−4.668^***^	<0.001
	PP	−0.001	0.006	−0.012–0.011	−0.136	0.893
	SP	−0.020	0.011	−0.042–0.002	−1.864	0.071

PP: primary psychopathy; SP: secondary psychopathy. There were 32 degrees of freedom for each effect. *p < 0.05; **p < 0.01; ***p < 0.001.

**Figure 1:**
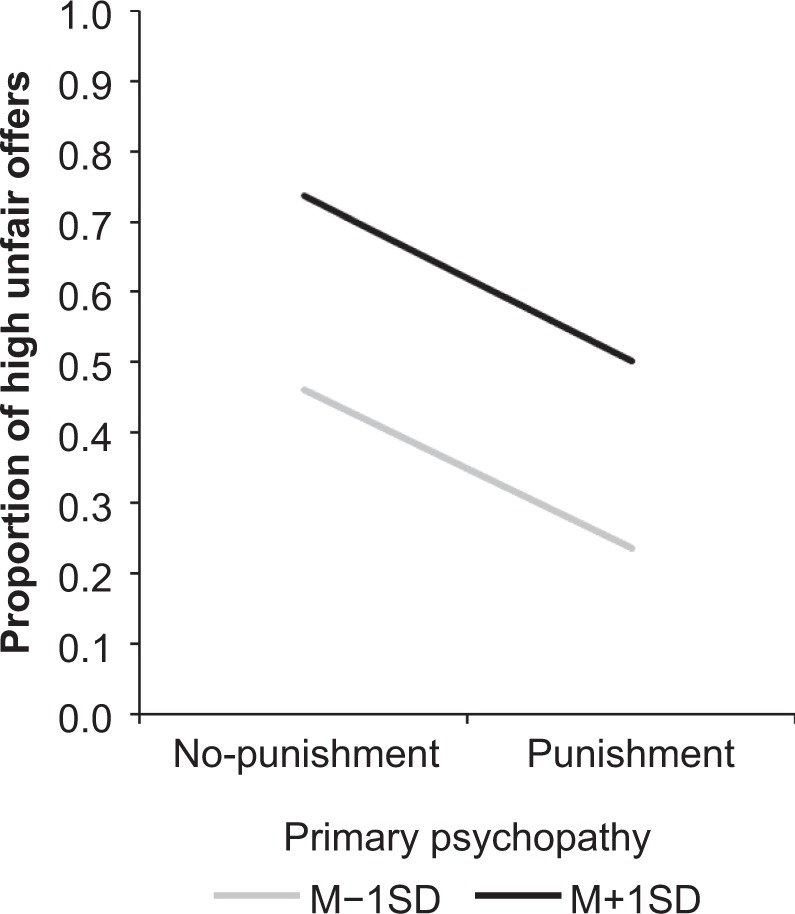
Modulation of the frequency of unfair offers as a function of the potential for punishment and primary psychopathy. The graph illustrates the result of simple slopes for the association between the potential for punishment and the frequency of high unfair offers according to primary psychopathy.

These results replicated the findings in a previous study^34^. Namely, as can be seen in Fig. 1, primary psychopathy was uniquely effective for increasing the frequency of high unfair offers in both the punishment and no-punishment conditions. However, the frequency of choosing such offers was decreased in response to a potential for punishment, regardless of the level of psychopathic traits. Similar results were found regarding the frequency of choosing relatively unfair offers between options (see Supplementary Table S2 and Fig. S1).

### Effects of punishment, unfairness, and psychopathic traits on skin conductance response

To examine whether psychopathic traits modulate the effects of punishment and unfairness on the SCR magnitude, HLM was used. The intraclass correlation coefficient for the SCR data was 0.81, which is an adequate effect size for analyzing the data using HLM. The results are shown in Table 3. Neither the potential for punishment (*B* = 0.009, *p* = 0.15) nor the level of unfairness (*B* = 0.0004, *p* = 0.87) had a significant main effect. In addition, the interaction of punishment and unfairness was not significant (*B* = 0.007, *p* = 0.22). On the other hand, there was a significant main effect of primary psychopathy, indicating that higher levels of primary psychopathy are associated with attenuated SCR magnitudes (*B* = −0.003, *p* = 0.02). However, no significant main effect of secondary psychopathy was found (*B* = −0.004, *p* = 0.18).

**Table 3:** Fixed effects for the HLM for predicting the magnitude of SCR.

Level 1	Level 2	Unstandardized coefficient	*SE*	95% CI	*t*-value	*p*-value
Intercept	Intercept	0.096	0.011	0.072–0.119	8.431^***^	<0.001
	PP	−0.003	0.001	−0.006–−0.001	−2.463^*^	0.019
	SP	−0.004	0.003	−0.009–0.002	−1.387	0.175
Punishment	Intercept	0.009	0.006	−0.003–0.021	1.484	0.148
	PP	0.0001	0.001	−0.002–0.002	0.178	0.860
	SP	−0.0002	0.002	−0.003–0.003	−0.145	0.886
Unfairness	Intercept	0.0005	0.003	−0.005–0.006	0.164	0.871
	PP	−0.001	0.0004	−0.001–0.0002	−1.452	0.156
	SP	0.0001	0.001	−0.001–0.002	0.193	0.848
Punishment × Unfairness	Intercept	0.007	0.006	−0.004–0.018	1.246	0.222
	PP	−0.002	0.001	−0.009–−0.0004	−2.912^***^	0.006
	SP	−0.0004	0.001	−0.003–0.003	−0.265	0.793

PP: primary psychopathy; SP: secondary psychopathy. There were 32 degrees of freedom for each effect. *p < 0.05; **p < 0.01; ***p < 0.001.

**Figure 2:**
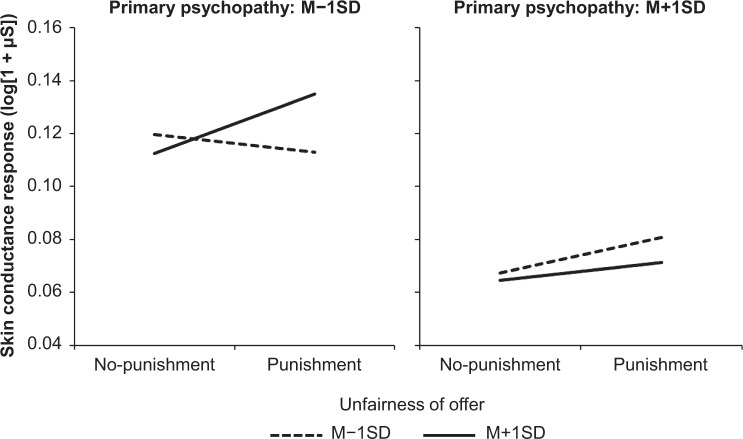
Modulation of the magnitude of SCR prior to the choice of offers as a function of the potential for punishment, unfairness of the offer, and primary psychopathy. The graph illustrates the result of simple slopes for the association between the potential for punishment and the magnitude of SCR according to the unfairness of offers and primary psychopathy.

The effect of primary psychopathy on the SCR magnitude was qualified by a significant cross-level interaction between punishment, unfairness, and primary psychopathy (*B* = −0.002, *p* = 0.01). Further analyses revealed a significant simple interaction between punishment and unfairness for participants with lower levels of primary psychopathy (*B* = 0.018, *p* = 0.003), but not for those who scored higher for primary psychopathy (*B* = −0.004, *p* = 0.60). As can be seen in Fig. 2, while the unfairness level of offers did not significantly affect the SCR magnitude in participants who scored lower for primary psychopathy when there was no potential for punishment (*B* = −0.004, *p* = 0.29), they exhibited increased SCR as a function of the unfairness of offers under the threat of punishment (*B* = 0.013, *p* = 0.01). In addition, when the unfairness level of an offer was higher, SCR magnitudes were larger in the punishment condition relative to the no-punishment condition (*B* = 0.023, *p* = 0.01). However, for participants who scored higher for primary psychopathy, the magnitude of SCR before choosing offers was not significantly modulated by the potential for punishment or the unfairness level (*ps* > 0.27).

Supplementary analyses revealed that participants who scored lower for primary psychopathy did not show a difference in SCR between contextually fair and unfair offers in the punishment condition. However, participants who scored higher for primary psychopathy exhibited smaller magnitudes of SCR when they would choose relatively unfair offers between options, compared to when they would choose relatively fair offers, despite a potential for punishment (see Supplementary Table S3 and Fig. S2A). On the other hand, in the no-punishment condition, the magnitude of SCR was increased prior to the choice of relatively unfair offers compared to relatively fair offers, regardless of any psychopathic tendencies (see Supplementary Table S4 and Fig. S2B).

### Mediation of the magnitude of skin conductance response between primary psychopathy and the choice of unfair offers

**Figure 3:**
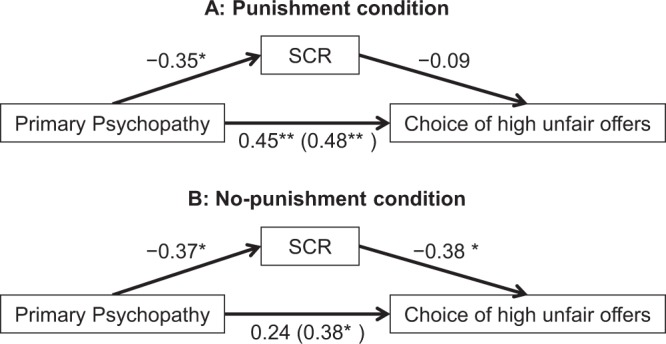
Mediation models. Illustrations show the indirect effect of SCR on the association between primary psychopathy and the choice of high unfair offers in (**A**) the punishment condition and (**B**) the no-punishment condition. *p < 0.05; **p < 0.01.

To examine whether SCR could explain the relationship between psychopathic traits and the choice of high unfair offers in the punishment and no-punishment conditions, mediation analyses^41^ with the bootstrap technique were conducted. For the punishment condition, a mediation analysis failed to demonstrate a significant indirect effect of primary psychopathy on the choice of high unfair offers through a reduced magnitude of SCR before the choice of such offers (*B* = 0.001, β = 0.032, *SE* = 0.002, BC CI = −0.0019–0.0063). As shown in Fig. 3A, higher levels of primary psychopathy significantly predicted increased frequencies of choosing high unfair offers, which carry a high risk for receiving punishment (*B* = 0.017, β = 0.484, *SE* = 0.005, *t*(33) = 3.179, *p* = 0.003). Primary psychopathy was also associated with reduced SCR magnitudes before the choice of such offers (*B* = −0.005, β = −0.349, *SE* = 0.002, *t*(33) = −2.141, *p* = 0.04). However, reduced SCR magnitudes did not significantly predict increased frequencies of high unfair offers after controlling for primary psychopathy (*B* = −0.251, β = −0.093, *SE* = 0.445, *t*(32) = −0.564, *p* = 0.58). The direct effect of primary psychopathy on the choice of high unfair offers remained statistically significant even after controlling for SCR (*B* = 0.016, β = 0.452, *SE* = 0.006, *t*(32) = 2.751, *p* = 0.01).

In contrast, for the no-punishment condition, as shown in Fig. 3B, the total effect of primary psychopathy on the decrease in the frequency of choosing highly unfair offers was significant (*B* = 0.019, β = 0.382, *SE* = 0.008, *t*(33) = 2.373, *p* = 0.02); however, after controlling for SCR, the direct effect of primary psychopathy on the choice of such offers was no longer significant (*B* = 0.012, β = 0.240, *SE* = 0.008, *t*(32) = 1.478, *p* = 0.15). Primary psychopathy significantly reduced SCR magnitudes before a choice of high unfair offers without incurring any risk of receiving punishment (*B* = −0.004, β = −0.370, *SE* = 0.002, *t*(33) = −2.285, *p* = 0.03). Moreover, the reduced SCR magnitude significantly predicted the increased frequencies of high unfair offers after controlling for primary psychopathy (*B* = −1.873, β = −0.385, *SE* = 0.789, *t*(32) = −2.373, *p* = 0.02). Consequently, the results of a mediation analysis indicated that the path from primary psychopathy to the choice of high unfair offers was significantly mediated by reduced SCR elicited prior to the choice of such offers in the no-punishment condition (*B* = 0.007, β = 0.142, *SE* = 0.005, BC CI = 0.0005–0.0202).

## Discussion

Consistent with a priori predictions, the present study demonstrated that primary psychopathy, but not secondary psychopathy, in a sub-clinical population was associated with attenuated magnitudes of SCR during decision-making regarding unfair monetary distribution to a partner with or without the potential for receiving punishment from the partner. It is difficult to simply interpret SCR findings because peripheral arousal is increased in response to both positive and negative stimuli^42^. Nevertheless, the results suggest the possibility that SCRs were increased under an increased potential for punishment. In particular, individuals who scored lower for primary psychopathy exhibited increased SCR as a function of the unfairness of offers under the threat of punishment. Moreover, they showed greater SCRs prior to high unfair offers in the punishment relative to no-punishment conditions. Such findings are compatible with a potential interpretation that SCRs prior to unfair offers in the punishment condition are related to defensive motivations. Interestingly, high tendencies in primary psychopathy moderated the potentiating effect of punishment on an SCR elicited prior to a choice of unfair offers. In addition, for participants who scored higher for primary psychopathy, the magnitude of SCR was reduced when they would choose relatively unfair offers between options, compared to when they would choose relatively fair offers. These results suggest that primary psychopathy reduces the sensitivity to anticipated punishment. Thus, the current findings support Lykken’s low-fear model of primary psychopathy, in light of the heterogeneity of psychopathic traits distributed in a continuum^43^.

Previous findings have shown that primary psychopathy attenuates physiological responses to threatening or aversive stimuli^7–10^. However, most of those studies were based on simple visual and acoustic stimuli, and it is unclear whether these findings extend to realistic social interactions. On the other hand, this study provides empirical data to support the idea that there is a deficit in the reactivity of the biological defensive system in primary psychopathy in a context closer to realistic social interactions. During the task, participants did not receive information on whether their offer had been accepted or rejected in each trial of the punishment condition. This experimental setting might raise a question about its compatibility with realistic interactions. However, especially when the partner who receives the offer is a stranger, individuals make an offer without knowing whether the partner will accept or reject an unfair offer. Therefore, to test risk sensitivity in an interaction with a stranger, the association between the level of unfairness and the probability of rejection had to be ambiguous throughout trials. In this sense, the current findings are of particular significance in terms of ecological validity.

Damasio’s SMH encourages the prediction that attenuated physiological arousal under the threat of punishment makes psychopathic individuals have low motivation to avoid high-risk options. In fact, primary psychopathy was associated with elevated frequencies of unfair offers that carry a high risk of receiving punishment, in line with previous findings regarding the association between primary psychopathy and increased self-interest behaviors in social decision-making tasks^34,44–46^ or increased risky choices in gambling tasks^47–49^. However, incompatible with this prediction, the present study failed to provide evidence for the mediating role of attenuated physiological arousal in the association between primary psychopathy and unfair offers when there was a potential for punishment. This null result of a mediation analysis suggests that increased norm violations as a function of primary psychopathy are less likely to be accounted for by a deficiency in physiological arousal in anticipation of punishment.

In the no-punishment condition, on the other hand, the current results indicated not only that primary psychopathy attenuated the magnitudes of SCR prior to unfair offers, but also that primary psychopathy was not associated with increased frequencies of unfair offers without the mediation of attenuated SCR magnitudes. Previous studies have found that primary psychopathy is associated with an increased unfairness of offers in the DG^31–34^, but these studies did not investigate physiological responses. The present study compensated for the lack of evidence that reduced physiological arousal is a key factor that accounts for the relationship between primary psychopathy and fairness norm violations.

The present findings highlight that whether or not attenuated physiological arousal mediates the relationship between primary psychopathy and unfair behavior is different between conditions. This difference is based on the result that anticipatory SCR did not predict the frequency of unfair offers after controlling for primary psychopathy in the punishment condition, in contrast to the no-punishment condition. Therefore, the bodily response may play a less decisive role in social decision-making under the threat of punishment. In support of this possibility, the frequency of unfair offers was decreased in response to the potential for punishment regardless of the tendency for primary psychopathy, even though punishment had no significant effect on the magnitude of SCR for participants who scored high for primary psychopathy. While focusing on individuals who scored lower for primary psychopathy, it seems that anticipatory SCRs predicted the level of unfairness of offers that they chose. However, even if larger magnitudes of SCR were evoked prior to the choice of unfair relative to fair offers, it is still questionable whether physiological arousal actually influences the decision-making process, in this condition at least. Rather, anticipatory SCRs might merely be increased in response to the potential for punishment.

The present study is inadequate to explain inconsistencies between electrodermal and behavioral results in the punishment condition. However, in line with the debate on the role of executive function in optimal decision-making under risk (e.g., in the IGT)^18^, findings from a neuroimaging study offer valuable insight into the role of executive function in the inhibitory control of self-interest unfair behavior under the threat of punishment^50,51^. In particular, the contrast between brain activations during decision-making regarding monetary distribution to a partner under the threat of punishment and those for punishment-free monetary distribution indicated the significance of prefrontal regions including the dorsolateral and ventrolateral prefrontal cortices, which have been shown to be reliably involved in goal maintenance and the inhibition of prepotent responses and self-interest behaviors^52–54^. These findings have important implications for the possibility that social decision-making under the threat of punishment is regulated under executive control.

On the other hand, where there is no risk of punishment, the emotions and motivations that spontaneously arise in each individual might modulate offers in a more direct way. Consistent with this possibility, a fairness preference corresponds closely to pleasure from fairness in the DG, but not in the UG^55^. In addition, unfairness aversion in the DG is theorized to be closely related to the feeling of guilt^35^. Following the SMH, it is assumed that physiological arousal that biases such emotions and motivations would place a priority on fairness over self-interest. In the present study, anticipatory SCRs in the no-punishment condition were not differentiated by the level of unfairness of offers that would be chosen, which is consistent with the finding that SCRs are increased regardless of affective valence^42^. This result appears to strengthen the doubt about the notion that physiological arousal directs individuals to a specific decision between options. However, if physiological arousal is related to affectively positive and negative properties of fairness and unfairness, respectively, it remains possible that it serves to prompt fair offers and to inhibit unfair offers. From that perspective, it is noteworthy that increased SCRs prior to the choice of unfair offers predicted decreased frequencies of the choice of such offers in the no-punishment condition. This result strengthens the possibility that even though there is no potential for punishment, physiological arousal plays a functional role in such internally-guided or preference-based decision-making. Therefore, the present findings suggest that primary psychopathy is associated with a deficit in somatic marker functioning that encourages spontaneous motivation of the inhibition of fairness norm violation.

Several limitations should be noted. First, the construct validity of the current task was unclear. Based on the task properties and findings from other studies, it was assumed that affective and executive functions would play a role in the task, but this study did not directly confirm their involvement. The subjective measurement of fearfulness and executive functions would have been useful to justify the implications of the present findings. Second, bodily responses in the present findings were restricted to the SCR as an index of physiological arousal. Because the SCR is not sensitive to the emotional valence of events, it is unclear whether such a measurement is enough to capture the putative somatic marker signaling if an option is good or bad. Measurement of a variety of other bodily responses including cardiac and muscle activities might provide more precise evidence for the role of bodily responses in the association between psychopathic traits and fairness norm violations. Third, the present study recruited participants from a non-incarcerated population and assessed psychopathic traits as continuums based on theoretical backgrounds. A growing body of evidence suggests that successful and unsuccessful subtypes of psychopathy have similarities and differences in cognitive, physiological, and neural characteristics^56–60^. Therefore, there are doubts about whether the current results would reflect those for psychopathy in criminal or clinical populations in practice. Future studies on the reduced utility of somatic markers as a function of psychopathy in not only successful, but also unsuccessful, populations could lead to a better understanding of the relationship between psychopathic traits and norm violations.

Taken together, the present findings may be useful for understanding the relationship among primary psychopathy, physiological arousal, and behavioral choices in social interactions. In seeking to explain how psychopathic traits are involved in social norm violations, there are limitations in any study on the relationships between any two of these variables. In particular, findings regarding the association between primary psychopathy and deficiencies in physiological arousal in anticipation of punishment stimuli support the low-fear model of primary psychopathy, but it is unclear whether such results indicate that there is a deficit in the functional role of bodily responses, as in the SMH. Instead, this study found that primary psychopathy was associated with both attenuated physiological arousal while participants decided to choose a fairness norm violation and an increased frequency of choosing fairness norm violations, regardless of whether or not the partner could take revenge. Furthermore, the results of mediation analyses provide suggestive evidence for the limits and potential of the explanation that primary psychopathy is associated with increased norm violations through a deficiency in physiological arousal. Further studies that focus on bodily responses will be encouraged to understand the biological mechanisms that underlie decision-making regarding social norm violations as a function of psychopathic traits.

## Methods

### Ethical statement

The study was approved by the review board of Hiroshima Shudo University and carried out in accordance with the Japanese ethical guidelines for medical and health research involving human subjects. All participants provided their written informed consent prior to participation in the experiment and were debriefed at the end of the experiment.

### Participants

Thirty-five Japanese undergraduate students (11 males) between 18 and 22 years of age (*M* = 19.23, *SD* = 1.22) participated in this experiment.

### Assessment of psychopathic traits

The participants completed a Japanese version^61^ of the LSRP^37^, which contains 26 items rated on a four-point Likert-type scale. These items are divided into two factors: primary and secondary psychopathy. The primary psychopathy subscale, which consists of 16 items, reflects manipulation, egocentricity and lack of empathy and remorse. In contrast, the secondary psychopathy subscale, which consists of 10 items, reflects impulsivity, stimulation-seeking and poor behavioral control. The total score of the LSRP in criminal populations has been found to be correlated with that of the Psychopathy Checklist-Revised (PCL-R)^2^, which suggests that the LSRP is, to some extent, measuring a construct related to the PCL-R diagnosed psychopathy^62^. The Japanese version of the LSRP was developed using back-translation for each item^61^; it has the same factor structure as the original version, and has been demonstrated to possess construct validity and adequate test-retest reliability^63^. Coefficient alphas in this study were 0.84 for primary psychopathy and 0.58 for secondary psychopathy. The alphas for secondary psychopathy on the LSRP in the previous and present studies are consistently low, but this is probably acceptable for a 10-item scale^34^. The ranges of scores were 22–57 (*M* = 35.80, *SD* = 7.09) for the primary psychopathy subscale, and 15–34 (*M* = 21.97, *SD* = 3.96) for the secondary psychopathy subscale, which suggest that the present study covered a wide spectrum of psychopathic traits. The average scores and variances were not dramatically different from other data in a recent study for a larger Japanese student sample (*N* = 348; primary psychopathy: *M* = 33.85, *SD* = 5.95; secondary psychopathy: *M* = 21.03, *SD* = 3.65)^34^.

### Experimental task and procedure

Participants were required to decide how to divide 10 Japanese yen between themselves and another person. They performed this task for 120 rounds, and thus they made decisions regarding a total of 1,200 yen (approximately 10 US dollars). Half of the 120 rounds were performed under the punishment condition; consistent with the rules of the UG, participants were instructed that they and their partner would receive money according to their offer if their partner accepted the offer, but neither party would receive any money in a given round if the partner rejected their offer in that round. The other rounds were conducted under the no-punishment condition; according to the rules of the DG, the partner would be unable to do anything but accept offers. Participant were also instructed that the partner was another student from the same university who would participate in the study after the current experiment. Thus, during the experiment, participants did not meet their partner face-to-face and were not informed whether their offers in the punishment condition had been accepted or rejected by the partner. In addition, participants were informed that they and the partner would be paid according to the results of their interactions. However, these instructions were a sham in practice because this study sought to investigate interpersonal decision-making based on a realistic situation. At the end of the experiment, participants were told that, in fact, there was no other student who played the role of the partner. All of the participants received 1,000 yen as compensation for participation regardless of their behavioral choices during the task.

**Figure 4:**
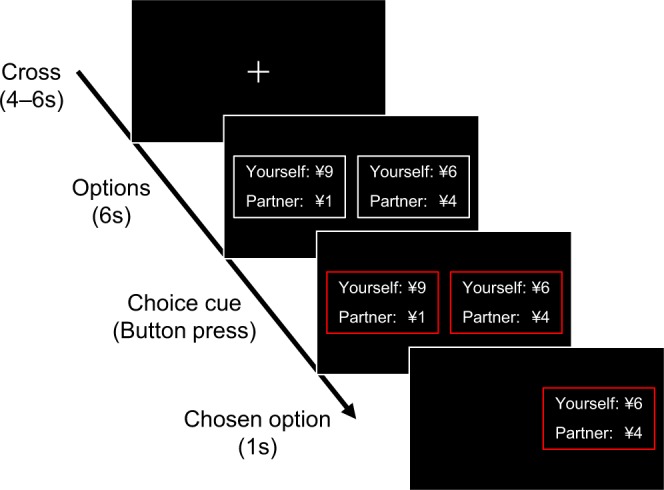
Example of a single round in the current monetary distribution task. The options were changed each round. The information displayed was the same in both the punishment and no-punishment conditions.

During the task, participants were seated in a reclining chair in front of a computer monitor. Once the task began, participants performed four sessions, each of which consisted of 30 rounds, under the punishment or no-punishment condition. The order of the punishment and no-punishment conditions was counterbalanced. Participants had a 3-min rest period between sessions. As shown in Fig. 4, in each round, participants chose between two potential offers, which were displayed on the left and right sides of the monitor (i.e., they could choose either the left option, which might be, for example, 9 yen to themselves and 1 yen to their partner, or the right option, which might be 6 yen to themselves and 4 yen to their partner). The amount offered to the partner ranged from 0 to 5 yen in 1-yen increments (0, 1, 2, 3, 4, 5 yen). The combinations of options were determined pseudo-randomly. For half of the rounds, participants had no option but to choose an offer (i.e., the left and right options were identical; for example, 7 yen to themselves and 3 yen to their partner). All combinations of options presented during the 60 rounds in each of the punishment and no-punishment conditions are shown in Supplementary Tables S5 and S6. The time course within a round was controlled using Presentation 18.1 software (Neurobehavioral Systems, Berkeley, CA). At the beginning of a round, a white-colored cross was presented for 4–6 s to allow autonomic activity to recover to baseline. Next, the two offer options, each of which was boxed by white-colored lines, were presented side-by-side for 6 s. When the color of the boxes turned red, the participant pressed the left or right button as fast as possible with their dominant hand to choose the option on the corresponding side. After this response, only the chosen option was presented on the monitor for 1 s.

After the experimental task, participants were requested to complete questionnaires of psychopathic traits, age and sex. In addition, participants were asked to respond to a brief questionnaire about the experimental setting using a visual analog scale from 0 to 100. The questionnaire consisted of four items: “(1) For cases where your partner could reject offers, how well did you understand the rule that if the partner chooses to reject the offer, neither you nor the partner will receive any money in that trial?”; “(2) For cases where your partner could not reject offers, how well did you understand the rule that both you and your partner will receive the amount of money determined by your offer?”; “(3) How strongly did you believe that you made offers to a real partner?”; and “(4) How strongly did you believe that you would receive real money according to the results of these trials?”

### Electrodermal data acquisition and reduction

Electrodermal activity (EDA) was obtained during the task at a constant 0.5 V using a psychophysiological monitoring system (MP36; Biopac Systems, Santa Barbara, CA). To record EDA, Ag/AgCl electrodes filled with gelled isotonic electrolyte were attached to the palmar and second phalanges of the index and middle fingers of the non-dominant hand. Electrodermal data were stored on a hard disc and analyzed offline using Acknowledge software (Biopac Systems, Santa Barbara, CA). Phasic changes in skin conductance were obtained from EDA data with a 0.05 Hz high-pass filter. The magnitude of the peak response was detected as the change from baseline to the peak of the response that began within 0.5–6.0 s after the options were displayed in each trial. The baseline was calculated as the mean during the 1 s prior to the onset of the options. Negative magnitudes were set to zero. Log transformation (log [1 + μS]) was used to normalize the distribution of SCR. Finally, the magnitudes were averaged with respect to each unfairness level of the offer (low: 5 and 4 yen; medium: 3 and 2 yen; high: 1 and 0 yen for the partner) for each of the punishment and no-punishment conditions.

### Analyses

For behavioral indices, the choice ratios of offers at each unfairness level were calculated based on rounds where participants could choose between offers with different unfairness levels in each of the punishment and no-punishment conditions. Analyses with hierarchical linear modeling (HLM) were conducted using HLM 7.01 software^64^. In HLM, first-level variables are nested within second-level variables. In addition, the first-level variables can be at the within-individual level of analysis, whereas the second-level variables can be at the between-individual level of analysis. Thus, in a model that predicts the choice of high unfair offers, the Level 1 variable was a repeated measure of the type of game reflecting whether participants chose offers under the threat of punishment or under no potential for punishment. On the other hand, the Level 2 variables were individual differences in the scores of primary and secondary psychopathy.

To examine whether the effect of unfairness on the SCR would be modulated by psychopathic traits in each punishment and no-punishment condition, an analysis with HLM was conducted. Repeated measures of the condition regarding the presence or absence of the potential for punishment, the unfairness of chosen offers, and their interaction were considered in Level 1, and primary and secondary psychopathy were considered in Level 2. In each analysis using HLM, since whether there was a potential for punishment was a categorical variable, effects coding was applied (no-punishment = −0.5, punishment = 0.5). The level of unfairness was treated as being continuous (low = 1, medium = 2, high = 3). All continuous variables (unfairness, primary psychopathy, and secondary psychopathy) were centered at the grand-mean before being introduced to the models. Random effects were hypothesized for all Level 1 variables. Next, main effects and the interactions of independent variables were assessed by estimating fixed effects with robust standard errors.

Mediation analyses with the bootstrap technique were conducted using the “PROCESS v2.16” macro^65^ for IBM SPSS Statistics software (IBM, Armonk, NY). Simple mediation can be accepted when an independent variable (X) affects a dependent variable (Y) through a potential intervening variable or mediator (M). In mediation analyses, primary psychopathy was inserted as X, the choice of high unfair offers in the punishment or no-punishment condition was inserted as Y, and the magnitude of SCR before a behavioral choice for a corresponding condition was inserted as M. All variables were mean-centered prior to entry into the model to facilitate the interpretation of coefficients. Cases in which secondary psychopathy would be inserted as X were not investigated because the total effect of secondary psychopathy on the choice of unfair offers could not be expected under either condition according to the results of correlations. The result of the indirect effect of X on Y through M was shown by a 95% bias-corrected confidence interval (BC CI) based on 10,000 bootstrap samples. If the 95% BC CI did not include zero, it was justified that the effect of X on Y decreased significantly after controlling for M.

## Supplementary information

Supplement

## Data Availability

The datasets generated during and/or analyzed during the current study are available from the corresponding author on reasonable request.
