# Supplementary material for: Mediating role of attenuated physiological arousal on the association between psychopathic traits and fairness norm violation
Source: Sci Rep. 2019 Dec 2;9:18053. doi: 10.1038/s41598-019-54676-z (PMC6889505; doi:10.1038/s41598-019-54676-z)
Supplement: Supplementary file 1 — Supplement [file 41598_2019_54676_MOESM1_ESM.pdf]

Supplementary information

Mediating role of attenuated physiological arousal on the association between psychopathic traits and fairness norm violation

Takahiro Osumi*

Department of Psychology, Hiroshima Shudo University, Hiroshima, Japan
* Corresponding author: tosumi@ncnp.go.jp

Zero-order correlations

Table S1 presents Pearson's correlation coefficients between psychopathic traits, choice ratios and SCR magnitudes for each type of offer in the punishment and no-punishment conditions. The significance of the Pearson correlations was reported without Bonferroni correction. Almost all of the correlations were no longer significant after Bonferroni correction.

Under a potential for punishment, higher scores of primary psychopathy were significantly associated with decreased frequencies of low unfair offers ($r = -0.53$, $p < 0.001$) and increased frequencies of high unfair offers ($r = 0.48$, $p = 0.003$). In addition, primary psychopathy was negatively associated with the magnitude of SCR when the unfairness level was medium ($r = -0.41$, $p = 0.02$) or high ($r = -0.35$, $p = 0.04$). On the other hand, secondary psychopathy was not significantly correlated with either behavioral choice or SCR for any unfairness level. Moreover, there were no significant correlations between the magnitude of SCR and the frequency of behavioral choices for any level of unfairness in the punishment condition.

On the other hand, when there was no threat of punishment, while a nearly significant negative correlation was found between primary psychopathy and the choice of low unfair offers ($r = -0.32$, $p = 0.06$), primary psychopathy was positively related to the choice of high unfair offers ($r = 0.38$, $p = 0.02$). Moreover, elevated levels of primary psychopathy were significantly associated with reduced amplitudes of SCR before low unfair ($r = -0.37$, $p = 0.03$) and high unfair offers ($r = -0.40$, $p = 0.03$). However, secondary psychopathy was not significantly correlated with either the choice of offers or SCR magnitudes before offers for any unfairness level. In the no-punishment condition, in contrast to the punishment condition, the positive correlation between the magnitude of SCR and the choice of low unfair offers was not statistically significant ($r = 0.30$, $p = 0.08$), but greater magnitudes of SCR were significantly related to lower frequencies of high unfair offers ($r = -0.47$, $p = 0.004$).

Effects of punishment and psychopathic traits on the choice of context-based unfair offers

An additional analysis was conducted on the ability of HLM to predict the choice of contextually unfair offers (relatively unfair offers between two options). The ratios of choosing relatively unfair offers between two options were calculated based on rounds where participants could choose between offers with different unfairness levels in each of the punishment and no-punishment conditions. In a model that predicts the choice of context-based unfair offers, the Level 1 variable was a repeated measure of the potential for punishment. On the other hand, the Level 2 variables were individual differences in the scores of primary and secondary psychopathy. Continuous variables (primary psychopathy and secondary psychopathy) were centered at the grand-mean before being introduced to the models. Random effects were hypothesized for all Level 1 variables. Next, main effects and the interactions of independent variables were assessed by estimating fixed effects with robust standard errors.

The ICC coefficient was 0.44, which supports a hierarchical structure. As shown in Table S2, the results of this HLM indicated main effects of punishment ($B = -0.196$, $p < 0.001$) and primary psychopathy ($B = 0.021$, $p < 0.001$). However, a main effect of secondary psychopathy was not found ($B = -0.005$, $p = 0.57$). There was no significant result for either an interaction between punishment and primary psychopathy ($B =$

0.002, p = 0.73) or an interaction between punishment and secondary psychopathy (B = −0.019, p = 0.09). Thus, as illustrated in Fig. S1, the frequency of choosing relatively unfair offers between options increased as a function of the tendency for primary psychopathy regardless of the potential for punishment. Nevertheless, the frequency of choosing such contextually unfair offers was decreased in response to the potential for punishment, independently of the psychopathic tendency.

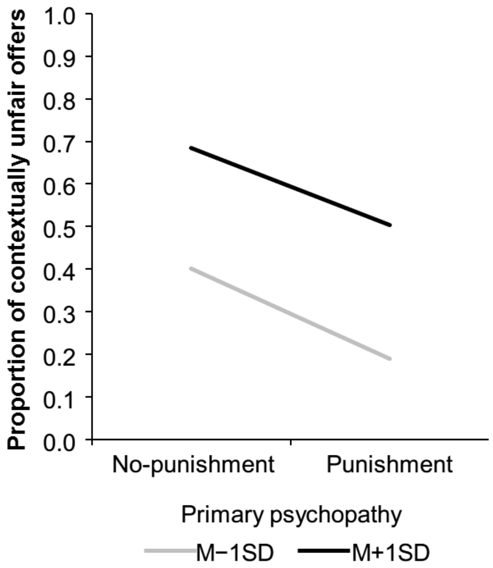

Fig. S1. Modulation of the frequency of choosing relatively unfair offers between options as a function of the potential for punishment and primary psychopathy. The graph illustrates the result of simple slopes for the association between the potential for punishment and the frequency of contextual unfair offers according to primary psychopathy.

Effects of context-based unfairness and psychopathic traits on skin conductance response in each punishment and no-punishment conditions

Supplementary analyses with HLM were designed to examine how a psychopathic tendency would modulate SCRs prior to the choice of contextually fair and unfair offers in the punishment and no-punishment conditions. The analyses for the punishment and no-punishment conditions were conducted separately, because the number of participants who chose both contextually fair and contextually unfair offers through rounds in either punishment or no-punishment condition was limited. In fact, the number of participants who were subjected to these analyses was 28 for the punishment condition, and 22 for the no-punishment condition. For these analyses, repeated measures of the potential for punishment, the type of chosen offers (relatively fair or relatively unfair offers between options) and their interaction were Level 1 variables, and primary and secondary psychopathy were Level 2 variables. Whether chosen offers were relatively fair or unfair between options was a categorical variable, so that effects coding was applied (relatively fair = −0.5, relatively unfair = 0.5). Continuous variables (primary psychopathy and secondary psychopathy) were centered at the grand-mean before being introduced to the models. Random effects were

hypothesized for Level 1 variable. Next, main effects and the interactions of independent variables were assessed by estimating fixed effects with robust standard errors.

The ICC coefficient for SCR data was 0.63 for the punishment condition and 0.75 for the no-punishment condition. As shown in Table S3, the results of the analysis with HLM for the punishment condition revealed a significant interaction between contextual unfairness and primary psychopathy ($B = -0.004$, $p = 0.03$). No other significant results were found. As illustrated in Fig. S2A, Participants who scored lower for primary psychopathy did not show a difference in SCR between contextually fair and unfair offers ($B = 0.007$, $p = 0.64$). However, participants who scored higher for primary psychopathy exhibited smaller magnitudes of SCR when they would choose relatively unfair offers between options, compared to when they would choose relatively fair offers, despite a potential for punishment ($B = -0.051$, $p = 0.03$). On the other hand, as shown in Table S4, an analysis with HLM for the no-punishment condition revealed only a significant main effect of contextual unfairness ($B = 0.029$, $p = 0.02$). The results mean that, as illustrated in Fig. S2B, the magnitude of SCR under no potential for punishment was increased prior to the choice of relatively unfair offers compared to relatively fair offers, regardless of any psychopathic tendencies.

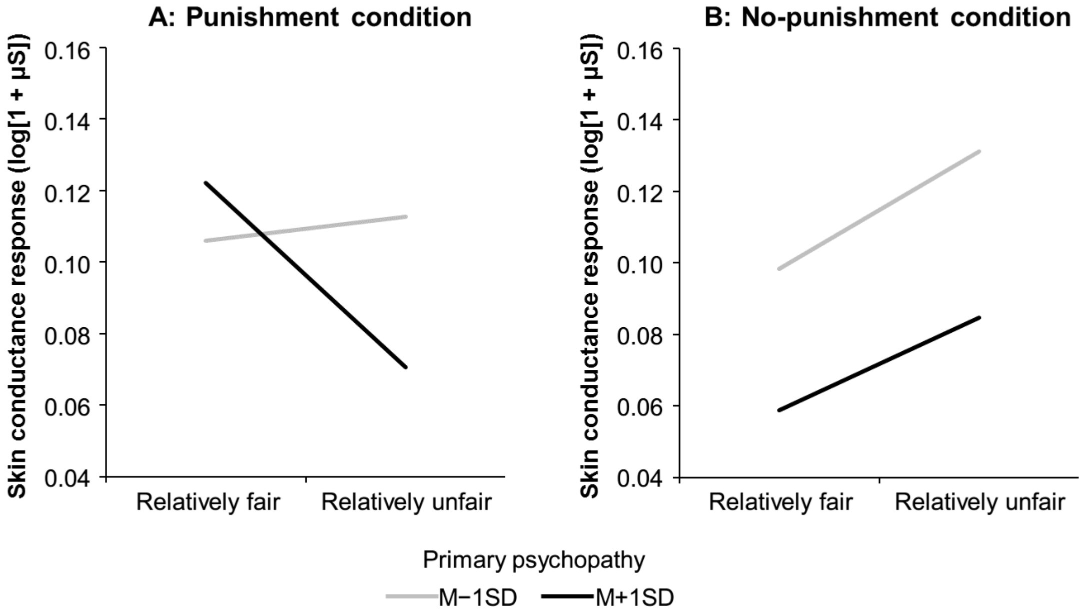

Fig. 2. Modulation of the magnitude of SCR prior to the choice of offers as a function of the contextual unfairness of the offer and primary psychopathy. The graph illustrates the result of simple slopes for the association between the contextual unfairness of offers and the magnitude of SCR according primary psychopathy in (A) the punishment condition and (B) the no-punishment condition.

			PP		SP		SCR	
			r	95% CI	*r*	95% CI	*r*	95% CI
Punishment	Low unfair offers	Choice	−0.53***	−0.74–−0.24	0.12	−0.22–0.44	0.13	−0.21–0.44
		SCR	−0.18	−0.49–0.16	−0.21	−0.51–0.13	-	-
	Medium unfair offers	Choice	0.19	−0.15–0.49	0.17	−0.18–0.47	−0.04	−0.36–0.30
		SCR	−0.41*	−0.65–−0.09	−0.26	−0.54–0.08	-	-
	High unfair offers	Choice	0.48**	0.18–0.70	−0.22	−0.51–0.13	−0.25	−0.54–0.09
		SCR	−0.35*	−0.61–−0.02	−0.20	−0.50–0.15	-	-
No-punishment	Low unfair offers	Choice	−0.32	−0.59–0.01	−0.12	−0.43–0.23	0.30	−0.04–0.57
		SCR	−0.36*	−0.62–0.03	−0.25	−0.54–0.09	-	-
	Medium unfair offers	Choice	−0.24	−0.54–0.10	0.19	−0.15–0.49	0.11	−0.23–0.43
		SCR	−0.30	−0.57–0.04	−0.15	−0.46–0.19	-	-
	High unfair offers	Choice	0.38*	0.06–0.63	0.07	−0.27–0.40	−0.47**	−0.70–−0.17
		SCR	−0.37*	−0.63–0.04	−0.25	−0.54–0.09	-	-

Table S1. Correlations between psychopathic traits and behavioral choice and anticipatory SCR. PP: primary psychopathy; SP: secondary psychopathy; SCR: skin conductance response. *** $p < .001$; ** $p < .01$; * $p < .05$ (two-tailed). Each *p*-values was uncorrected.

Level 1	Level 2	Unstandardized coefficient	*SE*	95% CI	*t*-value	*p*-value
Intercept	Intercept	0.445	0.044	0.356–0.534	10.179***	< 0.001
	PP	0.021	0.004	0.012–0.030	4.727***	< 0.001
	SP	−0.005	0.009	−0.024–0.014	−0.574	0.570
Punishment	Intercept	−0.196	0.053	−0.304–−0.089	−3.721***	< 0.001
	PP	0.002	0.006	−0.011–0.015	−0.355	0.725
	SP	−0.019	0.011	−0.042–0.003	−1.864	0.091

Table S2. Fixed effects for the HLM for predicting the choice of contextually unfair offers. PP: primary psychopathy; SP: secondary psychopathy. There were 32 degrees of freedom for each effect. * $p < .05$; ** $p < .01$; *** $p < .001$.

Level 1	Level 2	Unstandardized coefficient	*SE*	95% CI	*t*-value	*p*-value
Intercept	Intercept	0.103	0.015	0.071–0.135	6.651***	< 0.001
	PP	−0.001	0.002	−0.005–0.003	−0.451	0.656
	SP	−0.004	0.003	−0.011–0.002	−1.320	0.199
Contextual unfairness	Intercept	−0.022	0.013	−0.050–0.005	−1.679	0.106
	PP	−0.004	0.002	−0.008–−0.0003	−2.266*	0.032
	SP	0.007	0.004	−0.002–0.015	1.543	0.136

Table S3. Fixed effects for the HLM for predicting the magnitude of SCR by contextual unfairness of offers in the punishment condition. PP: primary psychopathy; SP: secondary psychopathy. There were 25 degrees of freedom for each effect. * $p < .05$; ** $p < .01$; *** $p < .001$.

Level 1	Level 2	Unstandardized coefficient	*SE*	95% CI	*t*-value	*p*-value
Intercept	Intercept	0.093	0.016	0.060–0.127	5.809***	< 0.001
	PP	−0.003	0.002	−0.008–0.002	−1.367	0.187
	SP	−0.003	0.003	−0.011–0.004	−0.975	0.342
Contextual unfairness	Intercept	−0.029	0.011	0.005–−0.053	2.565*	0.019
	PP	−0.0005	0.002	−0.005–0.004	−0.249	0.806
	SP	−0.003	0.003	−0.009–0.004	−0.892	0.383

Table S4. Fixed effects for the HLM for predicting the magnitude of SCR by contextual unfairness of offers in the no-punishment condition. PP: primary psychopathy; SP: secondary psychopathy. There were 19 degrees of freedom for each effect. * $p < .05$; ** $p < .01$; *** $p < .001$.

ID	Left option<br>Participant (yen)	Partner (yen)	Unfairness	Right option<br>Participant (yen)	Partner (yen)	Unfairness
1	10	0	High	9	1	High
2	10	0	High	8	2	Medium
3	10	0	High	7	3	Medium
4	10	0	High	6	4	Low
5	10	0	High	5	5	Low
6	9	1	High	10	0	High
7	9	1	High	8	2	Medium
8	9	1	High	7	3	Medium
9	9	1	High	6	4	Low
10	9	1	High	5	5	Low
11	8	2	Medium	10	0	High
12	8	2	Medium	9	1	High
13	8	2	Medium	7	3	Medium
14	8	2	Medium	6	4	Low
15	8	2	Medium	5	5	Low
16	7	3	Medium	10	0	High
17	7	3	Medium	9	1	High
18	7	3	Medium	8	2	Medium
19	7	3	Medium	6	4	Low
20	7	3	Medium	5	5	Low
21	6	4	Low	10	0	High
22	6	4	Low	9	1	High
23	6	4	Low	8	2	Medium
24	6	4	Low	7	3	Medium
25	6	4	Low	5	5	Low
26	5	5	Low	10	0	High
27	5	5	Low	9	1	High
28	5	5	Low	8	2	Medium
29	5	5	Low	7	3	Medium
30	5	5	Low	6	4	Low

Table S5. Offer options displayed on the left and right sides of the monitor in 30 rounds where the two displayed options were different. These options were common to both the punishment and no-punishment conditions.

ID	Left option<br>Participant (yen)	Partner (yen)	Unfairness	Right option<br>Participant (yen)	Partner (yen)	Unfairness
31	10	0	High	10	0	High
32	10	0	High	10	0	High
33	10	0	High	10	0	High
34	10	0	High	10	0	High
35	10	0	High	10	0	High
36	9	1	High	9	1	High
37	9	1	High	9	1	High
38	9	1	High	9	1	High
39	9	1	High	9	1	High
40	9	1	High	9	1	High
41	8	2	Medium	8	2	Medium
42	8	2	Medium	8	2	Medium
43	8	2	Medium	8	2	Medium
44	8	2	Medium	8	2	Medium
45	8	2	Medium	8	2	Medium
46	7	3	Medium	7	3	Medium
47	7	3	Medium	7	3	Medium
48	7	3	Medium	7	3	Medium
49	7	3	Medium	7	3	Medium
50	7	3	Medium	7	3	Medium
51	6	4	Low	6	4	Low
52	6	4	Low	6	4	Low
53	6	4	Low	6	4	Low
54	6	4	Low	6	4	Low
55	6	4	Low	6	4	Low
56	5	5	Low	5	5	Low
57	5	5	Low	5	5	Low
58	5	5	Low	5	5	Low
59	5	5	Low	5	5	Low
60	5	5	Low	5	5	Low

Table S6. Offer options displayed on the left and right sides of the monitor in 30 rounds where the two displayed options were the same. These options were common to both the punishment and no-punishment conditions.
